# Association between the degree of severity of COVID-19 infection during pregnancy and preterm premature rupture of membranes in a level III hospital in Peru

**DOI:** 10.17843/rpmesp.2023.404.12957

**Published:** 2023-12-18

**Authors:** Aitana Palma, Adrian Niño-Huertas, Guido Bendezu-Quispe, Percy Herrera-Añazco

**Affiliations:** 1 Universidad Peruana de Ciencias Aplicadas, Lima, Peru. Universidad Peruana de Ciencias Aplicadas Universidad Peruana de Ciencias Aplicadas Lima Peru; 2 Universidad Privada Norbert Wiener, Lima, Peru. Universidad Privada Norbert Wiener Universidad Privada Norbert Wiener Lima Peru; 3 Red Peruana de Salud Colectiva, Lima, Peru. Red Peruana de Salud Colectiva Lima Peru

**Keywords:** Severity of Illness Index, Coronavirus Infections, Premature Rupture of Fetal Membranes, Pregnant Women, Peru

## Abstract

**Objectives.:**

To determine the association between the degree of severity of COVID-19 infection during pregnancy and preterm premature rupture of membranes (PPROM) in a level III hospital in Peru.

**Materials and Methods.:**

Cross-sectional, analytical and observational study in women older than 18 years diagnosed with COVID-19 infection during pregnancy, between the years 2020 and 2022. Clinical and obstetric variables were collected. The chi-square and Fisher’s exact tests were used for the descriptive analysis. For the multivariate analysis, we calculated the prevalence ratio by using Poisson regression in crude and adjusted models. All statistical tests were performed considering a value of p<0.05 as significant and with a confidence level of 95%.

**Results.:**

We analyzed data from 163 pregnant women with COVID-19, of which 9.2% had PPROM; all were symptomatic cases. Mild COVID-19 cases were 1.10 times more likely to have PPROM (RPa=1.10; 95%CI: 1.02-1.18) and moderate/severe cases were 1.64 times more likely (RPa=1.64; 95%CI: 1.43-1.87), compared to asymptomatic cases.

**Conclusions.:**

We identified that a higher degree of severity of COVID-19 infection during pregnancy was associated with a higher probability of having PPROM.

## INTRODUCTION

In May 2023, the COVID-19 pandemic recorded more than 760 million positive cases and more than 6.9 million deaths worldwide ^1^. Peru was one of the countries most affected by the disease, having registered more than 4.5 million cases and more than 220 thousand deaths up to the same date ^1^. Several studies show that SARS-CoV-2 virus infection can cause multisystemic involvement in pregnant women with complications such as preterm delivery, fetal distress, premature rupture of membranes (PROM) and even maternal death ^2^. As in the rest of the world, COVID-19 also affected maternal health care services in Peru. In 2020, there were 30.7% more maternal deaths compared to 2019, and one in six of these deaths was associated with COVID-19 ^3^. PROM and preeclampsia were the most frequent obstetric complications and 14% of neonates had morbidities such as prematurity, low birth weight, sepsis and pneumonia ^4^.

Preterm premature rupture of membranes (PPROM) is the rupture of chorioamniotic membranes before the onset of labor in pregnancies of less than 37 weeks, with a prevalence of 3% to 4.5% of pregnancies globally ^5^^,^^6^. PPROM is associated with prematurity and its complications, such as neonatal respiratory distress syndrome and other problems derived from immature lungs, which are the main causes of perinatal morbidity and mortality ^5^. On the other hand, mothers with PPROM are susceptible to infections such as chorioamnionitis, endometritis and sepsis ^5^. In Peru, some studies prior to the pandemic found that PPROM was responsible for 20% of all perinatal deaths, with a prevalence of 4% to 18% of all deliveries, of which 50% were preterm ^7^.

These figures increased during the COVID-19 pandemic. A meta-analysis evaluating PPROM in women with COVID-19 found that the prevalence increased to 9.9% in pregnant women infected with the virus ^8^. Similarly, the Spanish Obstetric Emergency Group reported a higher incidence of PPROM in infected pregnant women (2.8%) compared to uninfected women (1.4%) ^9^. In Peru, during the state of health emergency, PPROM accounted for 5.6% of diagnoses at the National Maternal and Perinatal Institute (INMP), and its prevalence also increased during this period ^4^^,^^10^.

Infection and inflammation of the chorioamniotic membranes play a significant role in the development of PPROM. Inflammatory changes related to infection of the placenta and amniotic cavity (chorioamnionitis) frequently precede this complication ^11^. In this regard, factors related to the inflammatory state, including cytokine release, metalloproteinase activation and apoptosis have been documented in PPROM cases ^11^. COVID-19 infection causes dysregulation of the immune response, resulting in a dysfunctional protective response, leading to cytokine release syndrome with severe inflammation and multisystem failure ^12^. Indeed, by using immunohistochemical techniques, it was possible to detect inflammatory reactions in the placenta of women with viremia, which some authors suggest as a cause of chorioamnionitis ^13^.

In pregnant women infected with SARS-CoV-2, antigens and RNA of the virus were found on the fetal side of the placenta, and the placental tissue showed areas of inflammatory infiltrate ^13^^,^^14^. Likewise, the virus has been found in amniotic fluid and umbilical cord blood; however, these findings are not universal ^15^^,^^16^. Despite some discrepancies, it is possible to suggest that COVID-19 infection during pregnancy could allow passage of the virus into the placenta and amniotic cavity, producing inflammatory changes that increase the likelihood of PPROM. Additionally, it is possible that this association is conditioned by the severity of the infection, since, all patients were symptomatic in the report that found viral remnants, 34.9% required supplemental oxygen and 22.9% required intensive care ^15^. Finally, has been shown that plasma cytokine levels are higher in severe infections ^12^.

Although there is suggestive evidence of an association between COVID-19 infection and perinatal complications ^17^^-^^19^, there is little research specifically studying the relationship between the degree of infection severity in pregnancy and PPROM, particularly in Peru. Considering that PPROM can increase the frequency of perinatal complications, including maternal death ^5^, a study that evaluates this possible association may allow updating the management protocols, which should then be followed by health authorities in order to avoid this outcome and develop preventive strategies that contribute to the control of a complication that influences the maternal and neonatal morbidity and mortality rate. Therefore, this study aimed to determine the association between the degree of severity of COVID-19 infection during pregnancy and PPROM in a level III hospital in Peru.

**KEY MESSAGES**

Motivation for the study: The association between the degree of severity of COVID-19 with preterm premature rupture of membranes (PPROM) is likely, however, no studies have determined it.

Main findings: Pregnant women with mild COVID-19 infection were 1.10 times more likely to have PPROM and moderate/severe cases were 1.64 times more likely than asymptomatic cases.

Implications: These results reaffirm the need to prioritize the care of pregnant women according to the severity of COVID-19 infection in order to provide them with timely treatment.

## MATERIALS AND METHODS

### Design and Population

A cross-sectional, analytical and observational study was conducted. The population consisted of women over 18 years of age diagnosed with COVID-19 infection during pregnancy, whose delivery or fetal loss (from 22 weeks of gestation) occurred at the INMP during 2020-2022. Pregnant women diagnosed with COVID-19 by a positive molecular or antigenic test who were hospitalized in any of the INMP services were included. We also included patients who visited the hospital because of COVID-19 or who, while asymptomatic, were incidentally diagnosed with the infection during routine prenatal care. Pregnant women with a diagnosis of connective tissue disease, history of cervical conization, those who were transferred from another facility, and those with an incomplete medical history were excluded.

### Sample and sampling

We applied simple random probability sampling. Although this study sought to find a possible association between the degree of COVID-19 infection severity during pregnancy and the development of PPROM, this was an exploratory analysis since, at the time the protocol was designed, there was no history of studies that had evaluated the prevalence of PPROM according to each degree of infection severity. Therefore, in order to calculate the sample, we searched for studies that described the prevalence of COVID-19 infection during pregnancy ^10^, as well as the prevalence of PPROM in pregnant women diagnosed with COVID-19 ^20^. We compared independent proportions with the EPIDAT 4.2 tool. We applied the following parameters: population 1 (proportion of PPROM in pregnant women with COVID-19) was of 14.5% and population 2 (proportion of COVID-19 in pregnant women) was of 35.5%, with a confidence level of 95% and a power of 80%, obtaining a sample size of 132 participants. We considered 10% of losses due to incomplete medical records, 5% due to diagnosis of connective tissue diseases and 5% due to history of cervical conization, resulting in a minimum sample size of 163 participants.

### Variables

The independent variable was the degree of severity of COVID-19 infection during pregnancy and the dependent variable was PPROM. The former was categorized according to the “Clinical classification of COVID-19” established by the Ministry of Health (MINSA) as asymptomatic case, mild case, moderate case and severe case ^21^. This assessment was performed by the researchers according to the clinical presentation, laboratory results and imaging studies recorded by the treating physician in the patients’ medical records. PPROM was dichotomized into yes and no, defined according to its diagnosis and registration in the clinical history of the pregnant women.

Control variables included sociodemographic characteristics (age, socioeconomic level, and marital status), medical history (body mass index before pregnancy, history of tobacco use before pregnancy, history of drug use before pregnancy, and history of alcohol use before pregnancy), and obstetric-gynecologic history (anemia during pregnancy, ascending infections during pregnancy, vaginal bleeding during pregnancy, polyhydramnios, adequate prenatal control, number of products of conception, number of previous deliveries, history of previous preterm delivery, and history of fetal loss).

The data were collected from the medical records of the pregnant women and recorded individually by two study researchers (AP and ANH) in a data collection form prepared by them. The information collected was numerically coded and transferred to a self-generated database in Excel, without the use of personal identifiers.

### Statistical analysis

STATA v17 software was used for data analysis. Categorical variables were reported as absolute frequencies and percentages. The age variable was reported as median and interquartile range because it did not have a normal distribution. For bivariate analysis, the age variable was categorized into three: <20 years, 20-35 years and >35 years. We performed chi-square tests (no more than 20% of expected frequencies less than 5) and Fisher’s exact test (more than 20% of expected frequencies less than 5) since in all cases the possible association between two categorical variables was evaluated with unpaired samples. Finally, for the multivariate analysis, the “moderate case” and “severe case” categories of the variable “Degree of severity of COVID-19 infection during pregnancy” were grouped together, because the number of cases individually was very low. The prevalence ratio (PR) was calculated using Poisson regression in crude (cPR) and adjusted (aPR) models, choosing the variables for the adjusted models according to epidemiological criteria. For this purpose, a review of studies was carried out to determine the risk factors related to the development of PPROM ^5^^,^^22^, which were included as control variables within the gynecological-obstetric history, excluding those with low sample size (n<5). All statistical tests were performed considering a value of p<0.05 as significant and with a confidence level of 95%.

### Ethical Aspects

The research protocol was approved by the Ethics and Research Subcommittee of the Faculty of Health Sciences of the Peruvian University of Applied Sciences (UPC) by resolution No. FCS- SCEI/1146-11-21. It was also approved by the INMP through resolution N° 029-2022-CIEI/INMP. The research protocol was also registered in the Health Research Projects Platform (PRISA) of the National Health Institute (INS) with code EI00000003011.

## RESULTS

**Figure 1:**
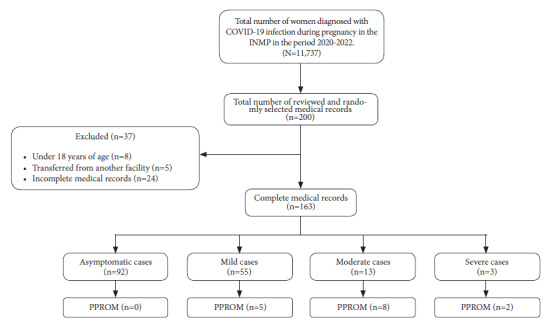
Flow chart of sample selection.

We selected and reviewed randomly 200 medical records out of a total of 11,737. Of all the selected records, eight of them belonged to patients under 18 years of age, five were from patients transferred from another facility and 24 were incomplete, so the final sample consisted of 163 medical records, which corresponds to the minimum sample size (Figure 1).

Regarding sociodemographic characteristics, the median age of pregnant women was 28 (IQR: 8) years, 48.5% belonged to the middle socioeconomic level and 71.8% were cohabitants. Regarding the medical records, 73.7% of the patients were overweight or obese and 11.6% had some harmful habit. Finally, the most frequent gynecological-obstetric condition was anemia during pregnancy (41.7%), 41.7% of the pregnant women had adequate prenatal controls, most of the patients had a single pregnancy (98.2%) and were multiparous (71.2%). The rest of the general characteristics are shown in Table 1.

**Table 1:** Association between preterm premature rupture of membranes and sociodemographic characteristics, medical history, obstetric-gynecologic history, and degree of severity of COVID-19 infection during pregnancy.

Variables	Total n=163 (%)	PPROM		p-value
		Yes n=15 (%)	No n=148 (%)	
Age (years)	28 (8) ^a^			
<20		0 (0.0)	3 (2.0)	0.107^b^
20-35		10 (66.7)	127 (85.8)	
>35		5 (33.3)	18 (12.2)	
Socioeconomic level				
Low	73 (44.8)	7 (46.7)	66 (44.6)	0.832^b^
Medium	79 (48.5)	8 (53.3)	71 (48.0)	
High	11 (6.7)	0 (0.0)	11 (7.4)	
Civil status				
No partner	17 (10.4)	1 (6.7)	16 (10.8)	1.000^b^
Married	29 (17.8)	3 (20.0)	26 (17.6)	
Cohabitant	117 (71.8)	11 (73.3)	106 (71.6)	
Body mass index before pregnancy				
Low weight (<18,5)	0 (0.0)	0 (0.0)	0 (0.0)	
Normal (18,5-24,9)	43 (26.4)	3 (20.0)	40 (27.0)	0.722^b^
Overweight (25-29,9)	86 (52.8)	8 (53.3)	78 (52.7)	
Obesity (≥30)	34 (20.9)	4 (26.7)	30 (20.3)	
History of tobacco use before pregnancy				
Yes	7 (4.3)	0 (0.0)	7 (4.7)	1.000^b^
No	156 (95.7)	15 (100.0)	141 (95.3)	
History of drug use prior to pregnancy				
Yes	2 (1.2)	0 (0.0)	2 (1.4)	1.000^b^
No	161 (98.8)	15 (100.0)	146 (98.6)	
History of alcohol consumption prior to pregnancy				
Yes	10 (6.1)	1 (6.7)	9 (6.1)	1.000^b^
No	153 (93.9)	14 (93.3)	139 (93.9)	
Anemia during pregnancy				
Yes	68 (41.7)	4 (26.7)	64 (43.2)	0.215^c^
No	95 (58.3)	11 (73.3)	84 (56.8)	
Ascending infections during pregnancy				
Yes	19 (11.7)	0 (0.0)	19 (12.8)	0.221^b^
No	144 (88.3)	15 (100.0)	129 (87.2)	
Vaginal bleeding during pregnancy				
Yes	12 (7.4)	0 (0.0)	12 (8.1)	0.605^b^
No	151 (92.6)	15 (100.0)	136 (91.9)	
Polyhydramnios				
Yes	4 (2.5)	0 (0.0)	4 (2.7)	1.000^b^
No	159 (97.5)	15 (100.0)	144 (97.3)	
Adequate prenatal care				
Yes	68 (41.7)	3 (20.0)	65 (43.9)	0.073^c^
No	95 (58.3)	12 (80.0)	83 (56.1)	
Number of products of conception				
Single pregnancy	160 (98.2)	15 (100.0)	145 (98.0)	1.000^b^
Multiple pregnancy	3 (1.8)	0 (0.0)	3 (2.0)	
Number of previous deliveries				
0 (nulliparous)	40 (24.5)	4 (26.7)	36 (24.3)	1.000^b^
1-3 (multiparous)	116 (71.2)	11 (73.3)	105 (71.0)	
≥4 (great multiparous)	7 (4.3)	0 (0.0)	7 (4.7)	
History of previous preterm delivery				
Yes	26 (16.0)	1 (6.7)	25 (16.9)	0.469^b^
No	137 (84.0)	14 (93.3)	123 (83.1)	
History of fetal loss				
Yes	47 (28.8)	5 (33.3)	42 (28.4)	0.766^b^
No	116 (71.2)	10 (66.7)	106 (71.6)	
Degree of severity of COVID-19 infection during pregnancy				
Asymptomatic case	92 (56.4)	0 (0.0)	92 (62.2)	<0.001^b^
Mild case	55 (33.7)	5 (33.3)	50 (33.8)	
Moderate case	13 (8.0)	8 (53.3)	5 (3.4)	
Severe case	3 (1.8)	2 (13.3)	1 (0.7)	

PPROM: preterm premature rupture of membranes.

a Median and interquartile range, ^b^ Fisher’s exact test, ^c^ Chi-square test.

We found that 9.2% of the patients had PPROM. Regarding the degree of severity of COVID-19 infection, most of the patients with PPROM were asymptomatic (56.4%) or mild cases (33.7%). Moderate and severe cases accounted for 8.0% and 1.8%, respectively (Table 1).

The bivariate analysis showed that all patients who developed PPROM were symptomatic, as 33.3% were mild cases, 53.3% moderate and 13.3% severe (p<0.001). In contrast, no statistically significant relationship was found between the study control variables and PPROM (Table 1).

The crude multivariate analysis showed that, with respect to asymptomatic cases, mild (cPR=1.09; 95%CI: 1.02-1.17) and moderate/severe cases (cPR=1.63; 95%CI: 1.40-1.88) were more likely to have PPROM. This association was also found during the multivariate model adjusted for anemia during pregnancy, ascending infections during pregnancy, vaginal bleeding during pregnancy, adequate prenatal control, number of previous deliveries, history of previous preterm delivery and history of fetal loss, where mild cases were 1.10 times more likely to have PPROM (aPR=1.10; 95%CI: 1.02-1.18) and moderate/severe cases had 1.64 times the probability (aPR=1.64; 95%CI: 1.43-1.87) compared to asymptomatic cases (Table 2).

**Table 2:** Association between degree of severity of COVID-19 infection during pregnancy and preterm premature rupture of membranes: crude and adjusted models.

Variables	cPR	95%CI	p-value	aPR^a^	95%CI	p-value
Degree of COVID-19 infection severity during pregnancy						
Asymptomatic case	Ref	-	-	Ref	-	-
Mild case	1.09	1.02‒1.17	0.015	1.10	1.02‒1.18	0.012
Moderate or severe case	1.63	1.40‒1.88	<0.001	1.64	1.43‒1.87	<0.001

cRP, crude prevalence ratio; aRP, adjusted prevalence ratio; 95%CI, 95% confidence interval.

a Model adjusted for: anemia during pregnancy, ascending infections during pregnancy, vaginal bleeding during pregnancy, adequate prenatal control, number of previous deliveries, history of previous preterm delivery, and history of fetal loss.

## DISCUSSION

Our main results show that about one in ten patients infected with COVID-19 developed PPROM. All of them were symptomatic cases. Pregnant women with mild cases were 1.10 times more likely to develop PPROM and those with moderate/severe cases were 1.64 times more likely compared to asymptomatic patients.

We found a PPROM frequency that is similar to other studies. A meta-analysis that included 16 cross-sectional studies and case series, reported that the frequency of PPROM in pregnant women infected with SARS-CoV-2 was 9.9% ^8^. Another systematic review of 39 studies, mostly retrospective studies and case series, showed that PPROM occurred in 8.9% of pregnancies with SARS-CoV-2 infection ^23^. In contrast, a systematic review of 19 studies, mainly case reports and retrospective studies, showed that PPROM occurred in 20.7% of patients infected with the virus ^24^. The different frequency rates between our results and other studies may be due to the fact that all the pregnant women we included were hospitalized with signs of severity, and the confidence interval of the mentioned frequency was wide, which would indicate a less precise estimation ^24^.

Our results suggest that the severity of COVID-19 infection is associated with a higher likelihood of complications during pregnancy, as other research has shown. A study involving 1219 pregnant women reported that adverse perinatal outcomes were more frequent in patients with more severe COVID-19 disease ^17^. Patients with severe/critical disease had an increased risk of cesarean section, hypertensive disorders of pregnancy, and preterm delivery compared to asymptomatic patients ^17^. Similarly, a systematic review of 62 studies, mostly cohorts, reported that patients with severe COVID-19 were at increased risk of developing gestational diabetes, preeclampsia, PROM (including PPROM), intrauterine growth restriction, fetal distress and placental abruption ^18^. In Peru, a study with similar results showed that the presence of symptoms of COVID-19 infection led to an increased risk of maternal complications as a composite variable, including PPROM, which was present in 25.9% of symptomatic cases and in only 9.5% of asymptomatic cases ^19^. Despite these similarities, this study specifically evaluates the association of PPROM, as a single variable, with all degrees of severity of infection, not only severe cases, which constitutes a contribution to the knowledge of this subject.

The development of PPROM after infection by COVID-19 can be explained by the presence of inflammatory changes in the chorioamniotic membranes, including the placenta, which usually precede the development of PPROM and represent findings frequently found in pregnant women with COVID-19 ^11^. The entry of the SARS-CoV-2 virus into human cells occurs through the ACE-2 receptor, also present in placental tissue; besides, viral particles and infiltration of inflammatory cells, mostly monocytes and neutrophils have been found during this process ^25^. Similarly, some studies have shown that SARS-CoV-2 infection can lead to significant histopathological changes in the placenta, the most frequent being maternal and fetal vascular malformations, with deficient placental perfusion, thrombus formation and villitis ^25^^,^^26^. In this regard, one study found that 25% of placentas from infected women were small for gestational age and 77% had poor perfusion characteristics ^27^. These findings suggest that COVID-19 may cause inadequate placental development, predisposing pregnant women to unfavorable maternal and fetal outcomes, including PPROM ^28^.

The relationship with the severity of the disease can be explained by some evidence showing that the concentrations of anti-SARS-CoV-2 (anti-Spike) antibodies, which trigger the inflammatory reaction in response to the presence of the virus, is higher in pregnant women with moderate to severe/critical disease ^28^. Thus, the greater development of placental abnormalities is directly proportional to the concentration of antibodies and severity of COVID-19, which is found in 57% of asymptomatic/mild cases, as opposed to 100% of moderate to severe cases ^28^. Likewise, the presence of more severe placental changes, including necrosis, were described in patients who required supplemental oxygen or intubation, i.e., more severe cases ^27^.

In the public health field, these results reaffirm the need to prioritize the care of pregnant women according to the degree of severity of COVID-19 infection in order to provide them with timely treatment. It also justifies administrative decisions that prioritize the allocation of resources to the care of pregnant women with the disease. Likewise, the data collected can be used as a preliminary study for future research related to this topic, especially taking into consideration that the results could change in the current context with a high rate of vaccination against COVID-19. As a matter of fact, a systematic review of nine studies found that vaccination in pregnant women reduces the probability of preterm births due to various causes, including PPROM ^29^.

Our study has some limitations. We did not find studies that evaluated the prevalence of PPROM according to each degree of severity of COVID-19 infection at the time this protocol was designed, therefore we used studies with similar proportions to calculate the sample. Furthermore, given that the number of participants ended up corresponding to the calculated minimum sample size and that there is a disproportion between the number of asymptomatic (n=92) and moderate/severe (n=16) cases, their comparison could constitute a clinical bias. However, due to the exploratory nature of this study, we consider our sample to be sufficient to raise hypotheses that should be verified in subsequent investigations. Similarly, there could have been selection bias due to the fact that we obtained data from a single center; however, the hospital we chose is a national referral center that receives pregnant women from different parts of Peru. There are other predisposing factors for PPROM that were not evaluated in this study because they correspond to information that is not usually found in medical records, such as the presence of nutritional deficiencies or a history of previous PPROM. For the same reason, other analytical data, such as gestational age at COVID-19 diagnosis and gestational age at the time of PPROM, could not be included. In addition, the medical records are handwritten, in a non-systematized and error-prone manner, so one must trust that the information has been completed properly. Also, it is possible that, at the beginning of the pandemic, some patients may not have been correctly diagnosed with the disease due to a lack of diagnostic tests, so there may be underreporting of pregnant women with COVID-19 and PPROM. Finally, COVID-19 disease has now evolved to a context where there is vaccination and control of the health emergency, which could have an effect on the current interpretation of the findings.

In conclusion, we found that a higher degree of severity of COVID-19 infection during pregnancy is associated with a greater probability of developing PPROM. Due to the exploratory nature of this study, we recommend further analogous research, with a larger sample size and representativeness, and taking into consideration the effect of vaccination and the current health context. Nevertheless, our study provides results that, due to their plausibility, merit consideration during decision making when evaluating a patient infected with COVID-19.
