# Supplementary material for: Association between the degree of severity of COVID-19 infection during pregnancy and preterm premature rupture of membranes in a level III hospital in Peru
Source: Rev Peru Med Exp Salud Publica. 2023 Dec 18;40(4):432–40. doi: 10.17843/rpmesp.2023.404.12957 (PMC11138821; doi:10.17843/rpmesp.2023.404.12957)
Supplement: Supplementary material. — Available in the electronic version of the RPMESP. [file rpmesp-40-04-12957-s001.pdf]

Material suplementario 1. Asociación entre el grado de severidad de la infección por COVID-19 durante el embarazo y la rotura prematura de membranas pretérmino: modelos crudos y ajustados de todas las variables del estudio.

Variables	RPc	IC95%	Valor de p	Rpa[a]	IC95%	Valor de p
Edad						
<20 años	Ref	-	-	Ref	-	-
20-35 años	1,07	1,03-1,12	0,001	1,03	0,98-1,10	0,257
>35 años	1,22	1,06-1,40	0,006	1,10	1,00-1,22	0,046
Nivel socioeconómico						
Bajo	Ref	-	-	Ref	-	-
Medio	1,00	0,92-1,10	0,912	0,98	0,92-1,04	0,418
Alto	0,91	0,86-0,97	0,004	0,98	0,92-1,04	0,409
Estado civil						
Sin pareja	Ref	-	-	Ref	-	-
Casada	1,04	0,90-1,21	0,580	0,97	0,87-1,09	0,627
Conviviente	1,03	0,92-1,16	0,582	0,98	0,89-1,09	0,758
Índice de masa corporal antes del embarazo						
Normal (18.5-24.9)	Ref	-	-	Ref	-	-
Sobrepeso (25-29.9)	1,02	0,93-1,12	0,643	1,01	0,93-1,10	0,789
Obesidad (≥30)	1,04	0,93-1,18	0,477	0,96	0,88-1,04	0,295
Antecedente de consumo de tabaco antes del embarazo						
Sí	0,91	0,87-0,95	<0,001	0,87	0,76-0,99	0,029
No	Ref	-	-	Ref	-	-
Antecedente de consumo de drogas antes del embarazo						
Sí	0,91	0,88-0,95	<0,001	0,99	0,94-1,04	0,612
No	Ref	-	-	Ref	-	-
Antecedente de consumo de alcohol antes del embarazo						
Sí	1,01	0,85-1,20	0,931	1,04	0,87-1,24	0,662
No	Ref	-	-	Ref	-	-
Anemia durante el embarazo						
Sí	0,95	0,88-1,03	0,190	0,98	0,92-1,04	0,484
No	Ref	-	-	Ref	-	-
Infecciones ascendentes durante el embarazo						
Sí	0,91	0,87-0,95	<0,001	0,91	0,84-0,98	0,016
No	Ref	-	-	Ref	-	-
Sangrado vaginal durante el embarazo						
Sí	0,91	0,87-0,95	<0,001	0,93	0,82-1,04	0,201
No	Ref	-	-	Ref	-	-
Polihidramnios						
Sí	0,91	0,88-0,95	<0,001	0,75	0,59-0,96	0,020
No	Ref	-	-	Ref	-	-
Control prenatal adecuado						
Sí	0,93	0,86-1,00	0,050	0,96	0,90-1,02	0,175
No	Ref	-	-	Ref	-	-
Número de productos de la concepción						
Embarazo único	Ref	-	-	Ref	-	-
Embarazo múltiple	0,91	0,88-0,95	<0,001	0,97	0,89-1,05	0,410
Número de partos previos						
0 (nulípara)	Ref	-	-	Ref	-	-
1-3 (multípara)	1,00	0,90-1,10	0,925	0,95	0,87-1,04	0,233
≥4 (gran multípara)	0,91	0,84-0,99	0,028	0,89	0,78-1,00	0,061
Antecedente de parto prematuro previo						
Sí	0,94	0,87-1,03	0,170	0,96	0,90-1,03	0,263
No	Ref	-	-	Ref	-	-
Antecedente de pérdida fetal						
Sí	1,02	0,93-1,12	0,697	0,98	0,90-1,07	0,725
No	Ref	-	-	Ref	-	-
Grado de severidad de la infección por COVID-19 durante el embarazo						
Caso asintomático	Ref	-	-	Ref	-	-
Caso leve	1,09	1,02-1,17	0,015	1,10	1,02-1,19	0,010
Caso moderado o severo	1,63	1,40-1,88	<0,001	1,65	1,45-1,89	<0,001

RPc: razón de prevalencia cruda; RPa: razón de prevalencia ajustada; IC95%: intervalo de confianza al 95%.
[a]Modelo ajustado por todas las variables del estudio.
