# Supplementary material for: Association between the degree of severity of COVID-19 infection during pregnancy and preterm premature rupture of membranes in a level III hospital in Peru
Source: Rev Peru Med Exp Salud Publica. 2023 Dec 18;40(4):432–40. doi: 10.17843/rpmesp.2023.404.12957 (PMC11138821; doi:10.17843/rpmesp.2023.404.12957)
Supplement: Translation

# Translation: Asociación entre el grado de severidad de la infección por COVID-19 durante el embarazo y la rotura prematura de membranas pretérmino en un hospital nivel III del Perú

**Authors:** Aitana Palma, Adrian Niño-Huertas, Guido Bendezu-Quispe, Percy Herrera-Añazco

**Affiliations:** 1 Universidad Peruana de Ciencias Aplicadas, Lima, Perú.; 2 Universidad Privada Norbert Wiener, Lima, Perú.; 3 Red Peruana de Salud Colectiva, Lima, Peru.

**Keywords:** Índice de Severidad de la Enfermedad, Infecciones por Coronavirus, Rotura Prematura de Membranas Fetales, Mujeres Embarazadas, Perú

## Abstract

**Objetivos.:**

Determinar la asociación entre el grado de severidad de la infección por COVID-19 durante el embarazo y la rotura prematura de membranas pretérmino (RPMP) en un hospital nivel III de Perú.

**Materiales y Métodos.:**

Estudio transversal, analítico y observacional en mujeres mayores de 18 años con diagnóstico de infección por COVID-19 en el embarazo durante el 2020-2022. Se recogieron variables clínicas y obstétricas. Para el análisis descriptivo se realizaron las pruebas de Chi Cuadrado y exacta de Fisher, y para el análisis multivariado, se calculó la razón de prevalencia mediante regresión de Poisson en modelos crudos y ajustados. Todas las pruebas estadísticas se realizaron considerando un valor de p<0,05 como significativo y con un nivel de confianza de 95%.

**Resultados.:**

Se analizaron los datos de 163 gestantes con COVID-19, de las cuales el 9,2% tuvieron RPMP, todas fueron casos sintomáticos. Los casos leves de COVID-19 tuvieron 1,10 veces la probabilidad de presentar RPMP (RPa=1,10; IC95%: 1,02−1,18) y los casos moderados/severos tuvieron 1,64 veces esta probabilidad (RPa=1,64; IC95%: 1,43−1,87), en comparación con los casos asintomáticos.

**Conclusiones.:**

Se identificó que un mayor grado de severidad de la infección por COVID-19 durante el embarazo se asoció a una mayor probabilidad de tener RPMP.

## INTRODUCCIÓN

En mayo del 2023, la pandemia de la COVID-19 registró más de 760 millones de casos positivos y más de 6,9 millones de muertes a nivel mundial ^1^. El Perú fue uno de los países más afectados por la enfermedad, habiendo registrado más de 4,5 millones de casos y más de 220 mil fallecidos hasta la misma fecha ^1^. Diversos estudios demuestran que la infección por el virus del SARS-CoV-2 puede generar un compromiso multisistémico en la gestante con complicaciones como el parto pretérmino, distress fetal, rotura prematura de membranas (RPM) e incluso la muerte materna ^2^. Al igual que en el resto del mundo, en Perú, la COVID-19 también afectó la atención en los servicios de salud materna. En el 2020, se produjeron un 30,7% más muertes maternas con respecto al 2019 y una de cada seis de estas muertes estuvo asociada a la COVID-19 ^3^. La RPM y la preeclampsia fueron las complicaciones obstétricas más frecuentes y el 14% de los neonatos presentaron morbilidades como prematuridad, bajo peso al nacer, sepsis y neumonía ^4^.

La rotura prematura de membranas pretérmino (RPMP) es la rotura de membranas corioamnióticas antes del inicio del trabajo de parto en embarazos menores a las 37 semanas de gestación, con una prevalencia de 3% a 4,5% de embarazos a nivel global ^5^^,^^6^. La RPMP se asocia a la prematuridad y sus complicaciones, tales como el síndrome de dificultad respiratoria neonatal y otros problemas derivados de pulmones inmaduros, las que constituyen las principales causas de morbimortalidad perinatal ^5^. Por otro lado, las madres con RPMP resultan susceptibles a infecciones como la corioamnionitis, endometritis y sepsis ^5^. En el Perú, algunos estudios previos a la pandemia encontraron que la RPMP era responsable del 20% de todas las muertes perinatales, con una prevalencia del 4% al 18% de todos los partos, de los que el 50% eran pretérmino ^7^.

Durante la pandemia de la COVID-19 estas cifras se incrementaron. Un metaanálisis que evaluó la RPMP en mujeres con la COVID-19 encontró que su prevalencia aumentó a 9,9% en embarazadas infectadas por el virus ^8^. De igual forma, el Spanish Obstetric Emergency Group demostró una mayor incidencia de RPMP en gestantes infectadas (2,8%) en comparación con las no infectadas (1,4%) ^9^. En Perú, durante el estado de emergencia sanitaria, la RPMP representó el 5,6% de diagnósticos en el Instituto Nacional Materno Perinatal (INMP), y su prevalencia también aumentó durante este periodo ^4^^,^^10^.

La infección e inflamación de las membranas corioamnióticas desempeñan un papel significativo en el desarrollo de la RPMP. Los cambios inflamatorios relacionados a la infección de la placenta y cavidad amniótica (corioamnionitis) frecuentemente preceden a esta complicación ^11^. En ese sentido, factores relacionados al estado inflamatorio, incluidos la liberación de citoquinas, la activación de metaloproteinasas y la apoptosis fueron documentadas en la RPMP ^11^. La infección por COVID-19 genera una desregulación de la respuesta inmune, lo que produce una respuesta protectora disfuncional, provocando el síndrome de liberación de citoquinas con inflamación severa y falla multisistémica ^12^. En efecto, mediante el uso de técnicas de inmunohistoquímica, se logró registrar la presencia de reacciones inflamatorias en la placenta de mujeres con viremia, lo que algunos autores sugieren como causa de la corioamnionitis ^13^.

En gestantes infectadas con el SARS-CoV-2 se encontraron antígenos y ARN del virus en el lado fetal de la placenta y el tejido placentario manifestó áreas de infiltrado inflamatorio ^13^^,^^14^. Asimismo, se ha encontrado la presencia del virus en el líquido amniótico y sangre del cordón umbilical, no obstante, estos hallazgos no son universales ^15^^,^^16^. A pesar de las discrepancias, es posible sugerir que la infección por COVID-19 durante el embarazo podría permitir el paso del virus a la placenta y la cavidad amniótica, produciendo cambios inflamatorios que aumentan la probabilidad de RPMP. Adicionalmente, es posible que esta asociación esté condicionada por la severidad de la infección, ya que, en el reporte que encontró restos virales, todas las pacientes eran sintomáticas, el 34,9% requirió oxígeno suplementario y el 22,9% cuidados intensivos ^15^. Por último, se demostró que los niveles plasmáticos de citoquinas son más elevados en infecciones severas ^12^.

A pesar de que existe evidencia sugerente de la asociación entre la infección por COVID-19 y complicaciones perinatales ^17^^-^^19^, se encontraron escasas investigaciones que estudien específicamente la relación entre el grado de severidad de la infección en el embarazo y la RPMP, sobre todo en Perú. Considerando que la RPMP puede aumentar la frecuencia de complicaciones perinatales, incluida la muerte materna ^5^, un estudio sobre el tema que demuestre esta posible asociación permitirá actualizar los protocolos de manejo que deben seguir las autoridades sanitarias para evitar este desenlace y desarrollar estrategias preventivas que contribuyan al control de una complicación que influye sobre la tasa de morbimortalidad materna y neonatal. Por lo expuesto, el objetivo de la presente investigación fue determinar la asociación entre el grado de severidad de la infección por COVID-19 durante el embarazo y la RPMP en un hospital nivel III de Perú.

**MENSAJE CLAVE**

Motivación para realizar el estudio: Es factible la asociación entre el grado de severidad de la COVID-19 con la rotura prematura de membranas pretérmino (RPMP), no obstante, no hay estudios que la hayan determinado.

Principales hallazgos: Las gestantes con infección leve por COVID-19 tuvieron 1,10 veces la probabilidad de tener RPMP y los casos moderados/severos tuvieron 1,64 veces esta probabilidad en comparación con los casos asintomáticos.

Implicancias: Estos resultados reafirman la necesidad de priorizar la atención de gestantes según el grado de severidad de la infección por COVID-19 para poder brindarles un tratamiento oportuno.

## MATERIALES Y MÉTODOS

### Diseño y Población

Se realizó un estudio transversal, analítico y observacional. La población estuvo constituida por mujeres mayores de 18 años con diagnóstico de infección por COVID-19 durante el embarazo, cuyo parto o pérdida fetal (a partir de las 22 semanas de gestación) fue en el INMP durante el 2020-2022. Se incluyeron a gestantes diagnosticadas de la COVID-19 mediante una prueba molecular o antigénica positiva que fueron hospitalizadas en cualquiera de los servicios del INMP. Asimismo, se incluyeron a las pacientes que acudieron al hospital a causa de la COVID-19 o a las que, estando asintomáticas, se les diagnosticó incidentalmente con la infección durante el control prenatal rutinario. Se excluyeron a las gestantes con diagnóstico de enfermedades del tejido conectivo, con antecedentes de conización cervical, a las que fueron trasladadas de otro establecimiento y las que tenían una historia clínica incompleta.

### Muestra y muestreo

El muestreo fue probabilístico aleatorio simple. Si bien el presente estudio buscó encontrar una posible asociación entre el grado de severidad de la infección por COVID-19 durante el embarazo y el desarrollo de RPMP, este se trató de un análisis exploratorio ya que, en el momento de diseñar el protocolo, no se encontraron antecedentes de estudios que hayan evaluado la prevalencia de RPMP según cada grado de severidad de la infección. Por lo tanto, para realizar el cálculo de la muestra, se buscaron estudios que describieron la prevalencia de la infección por COVID-19 en la gestación ^10^, al igual que la prevalencia de la RPMP en gestantes con diagnóstico de la COVID-19 ^20^. Utilizando la herramienta EPIDAT 4,2, se realizó una comparación de proporciones independientes. Los parámetros utilizados para el cálculo fueron: población 1 (proporción de RPMP en gestantes con la COVID-19) de 14,5% y población 2 (proporción de la COVID-19 en gestantes) de 35,5%, con un nivel de confianza de 95% y una potencia de 80%, obteniendo un tamaño de muestra de 132 participantes. Se consideró 10% de pérdidas por historias clínicas incompletas, 5% por diagnóstico de enfermedades del tejido conectivo y 5% por antecedentes de conización cervical, obteniendo finalmente un tamaño mínimo de muestra de 163 participantes.

### Variables

La variable independiente fue el grado de severidad de la infección por COVID-19 durante el embarazo y la variable dependiente fue la RPMP. La primera fue categorizada según la «Clasificación clínica de COVID-19» establecida por el Ministerio de Salud (MINSA) como caso asintomático, caso leve, caso moderado y caso severo ^21^. Esta valoración fue realizada por los investigadores de acuerdo con la presentación clínica, valores de laboratorio y estudios de imágenes registrados por el médico tratante en las historias clínicas de las pacientes. La RPMP fue dicotomizada en sí y no, definida según su diagnóstico y registro en la historia clínica de las gestantes.

Las variables de control incluyeron las características sociodemográficas (edad, nivel socioeconómico y estado civil), antecedentes médicos (índice de masa corporal antes del embarazo, antecedente de consumo de tabaco antes del embarazo, antecedente de consumo de drogas antes del embarazo, y antecedente de consumo de alcohol antes del embarazo) y antecedentes gineco-obstétricos (anemia durante el embarazo, infecciones ascendentes durante el embarazo, sangrado vaginal durante el embarazo, polihidramnios, control prenatal adecuado, número de productos de la concepción, número de partos previos, antecedente de parto prematuro previo y antecedente de pérdida fetal).

Los datos fueron recogidos de las historias clínicas de las gestantes y registrados individualmente por los dos investigadores del estudio (AP y ANH) en la ficha de recolección de datos, elaborada por los mismos. La información recolectada fue codificada numéricamente y trasladada a una base de datos autogenerada en Excel, sin el uso de identificadores personales.

### Análisis estadístico

Para el análisis de los datos se utilizó el software STATA v17. Para la descripción de las variables, las categóricas se reportaron en frecuencias absolutas y porcentajes. La variable de edad se reportó a través de la mediana y el rango intercuartil por no seguir la distribución normal. Para el análisis bivariado, la variable de edad se categorizó en tres valores finales: <20 años, 20-35 años y >35 años. Dado que en todos los casos se evaluó la posible asociación entre dos variables categóricas con muestras no pareadas, se realizaron las pruebas de chi cuadrado (no más del 20% de frecuencias esperadas menores que 5) y la prueba exacta de Fisher (más del 20% de frecuencias esperadas menores que 5). Finalmente, para el análisis multivariado, se agruparon las categorías de «caso moderado» y «caso severo» de la variable de «Grado de severidad de la infección por COVID-19 durante el embarazo», debido a que el número de casos de forma individual era muy bajo. Se calculó la razón de prevalencia (RP), mediante regresión de Poisson en modelos crudos (RPc) y ajustados (RPa), escogiendo las variables para los modelos ajustados según criterio epidemiológico. Para ello, se realizó una revisión de estudios que determinó los factores de riesgo relacionados al desarrollo de RPMP ^5^^,^^22^, los cuales fueron incluidos como variables de control dentro de los antecedentes gineco-obstétricos, excluyendo aquellas con bajo tamaño muestral (n<5). Todas las pruebas estadísticas se realizaron considerando un valor de p<0,05 como significativo y con un nivel de confianza del 95%.

### Aspectos éticos

El protocolo de investigación fue aprobado por el Subcomité de Ética e Investigación de la Facultad de Ciencias de la Salud de la Universidad Peruana de Ciencias Aplicadas (UPC) mediante resolución Nº FCS- SCEI/1146-11-21. También fue aprobado por el INMP mediante resolución N° 029-2022-CIEI/INMP. Asimismo, se registró en la Plataforma de Proyectos de Investigación en Salud (PRISA) del Instituto Nacional de Salud (INS) con el código EI00000003011.

## RESULTADOS

De un total de 11 737 historias clínicas, 200 fueron seleccionadas y revisadas aleatoriamente. De todas las historias seleccionadas, ocho de ellas pertenecieron a menores de 18 años, cinco a pacientes trasladadas de otro establecimiento y 24 estuvieron incompletas, por lo que la muestra final estuvo constituida por 163 historias clínicas, que corresponde al tamaño mínimo de muestra (Figura 1).

**Figura 1:** Flujograma de la selección de la muestra.

Con respecto a las características sociodemográficas, la mediana de edad de las gestantes fue de 28 (RIC: 8) años, el 48,5% pertenecieron al nivel socioeconómico medio y el 71,8% eran convivientes. En relación con los antecedentes médicos, el 73,7% de las pacientes presentó sobrepeso u obesidad y el 11,6% contó con algún hábito nocivo. Finalmente, en lo referente a los antecedentes gineco-obstétricos, el más frecuente fue la anemia durante el embarazo (41,7%), el 41,7% de gestantes tuvo un control prenatal adecuado, la mayoría de las pacientes presentó un embarazo único (98,2%) y correspondieron al grupo de multíparas (71,2%). El resto de las características generales se muestran en la Tabla 1.

**Tabla 1:** Asociación entre la rotura prematura de membranas pretérmino y las características sociodemográficas, antecedentes médicos, antecedentes gineco-obstétricos y grado de severidad de la infección por COVID-19 durante el embarazo.

Variables	Total n=163 (%)	RPMP		Valor de p
		Sí n=15 (%)	No n=148 (%)	
Edad (años)	28 (8)^a^			
<20		0 (0,0)	3 (2,0)	0,107^b^
20-35		10 (66,7)	127 (85,8)	
>35		5 (33,3)	18 (12,2)	
Nivel socioeconómico				
Bajo	73 (44,8)	7 (46,7)	66 (44,6)	0,832^b^
Medio	79 (48,5)	8 (53,3)	71 (48,0)	
Alto	11 (6,7)	0 (0,0)	11 (7,4)	
Estado civil				
Sin pareja	17 (10,4)	1 (6,7)	16 (10,8)	1,000^b^
Casada	29 (17,8)	3 (20,0)	26 (17,6)	
Conviviente	117 (71,8)	11 (73,3)	106 (71,6)	
Índice de masa corporal antes del embarazo				
Bajo peso (<18,5)	0 (0,0)	0 (0,0)	0 (0,0)	
Normal (18,5-24,9)	43 (26,4)	3 (20,0)	40 (27,0)	0,722^b^
Sobrepeso (25-29,9)	86 (52,8)	8 (53,3)	78 (52,7)	
Obesidad (≥30)	34 (20,9)	4 (26,7)	30 (20,3)	
Antecedente de consumo de tabaco antes del embarazo				
Sí	7 (4,3)	0 (0,0)	7 (4,7)	1,000^b^
No	156 (95,7)	15 (100,0)	141 (95,3)	
Antecedente de consumo de drogas antes del embarazo				
Sí	2 (1,2)	0 (0,0)	2 (1,4)	1,000^b^
No	161 (98,8)	15 (100,0)	146 (98,6)	
Antecedente de consumo de alcohol antes del embarazo				
Sí	10 (6,1)	1 (6,7)	9 (6,1)	1,000^b^
No	153 (93,9)	14 (93,3)	139 (93,9)	
Anemia durante el embarazo				
Sí	68 (41,7)	4 (26,7)	64 (43,2)	0,215^c^
No	95 (58,3)	11 (73,3)	84 (56,8)	
Infecciones ascendentes durante el embarazo				
Sí	19 (11,7)	0 (0,0)	19 (12,8)	0,221^b^
No	144 (88,3)	15 (100,0)	129 (87,2)	
Sangrado vaginal durante el embarazo				
Sí	12 (7,4)	0 (0,0)	12 (8,1)	0,605^b^
No	151 (92,6)	15 (100,0)	136 (91,9)	
Polihidramnios				
Sí	4 (2,5)	0 (0,0)	4 (2,7)	1,000^b^
No	159 (97,5)	15 (100,0)	144 (97,3)	
Control prenatal adecuado				
Sí	68 (41,7)	3 (20,0)	65 (43,9)	0,073^c^
No	95 (58,3)	12 (80,0)	83 (56,1)	
Número de productos de la concepción				
Embarazo único	160 (98,2)	15 (100,0)	145 (98,0)	1,000^b^
Embarazo múltiple	3 (1,8)	0 (0,0)	3 (2,0)	
Número de partos previos				
0 (nulípara)	40 (24,5)	4 (26,7)	36 (24,3)	1,000^b^
1-3 (multípara)	116 (71,2)	11 (73,3)	105 (71,0)	
≥4 (gran multípara)	7 (4,3)	0 (0,0)	7 (4,7)	
Antecedente de parto prematuro previo				
Sí	26 (16,0)	1 (6,7)	25 (16,9)	0,469^b^
No	137 (84,0)	14 (93,3)	123 (83,1)	
Antecedente de pérdida fetal				
Sí	47 (28,8)	5 (33,3)	42 (28,4)	0,766^b^
No	116 (71,2)	10 (66,7)	106 (71,6)	
Grado de severidad de la infección por COVID-19 durante el embarazo				
Caso asintomático	92 (56,4)	0 (0,0)	92 (62,2)	<0,001^b^
Caso leve	55 (33,7)	5 (33,3)	50 (33,8)	
Caso moderado	13 (8,0)	8 (53,3)	5 (3,4)	
Caso severo	3 (1,8)	2 (13,3)	1 (0,7)	

RPMP: rotura prematura de membranas pretérmino.

a Mediana y rango intercuartil, ^b^ prueba exacta de Fisher, ^c^ prueba de Chi cuadrado.

El 9,2% de las pacientes presentó RPMP. Respecto al grado de severidad de la infección por COVID-19, la mayoría de las pacientes con RPMP fueron casos asintomáticos (56,4%) o leves (33,7%). Los casos moderados y severos representaron el 8,0% y el 1,8%, respectivamente (Tabla 1).

El análisis bivariado demostró que todas las pacientes que desarrollaron RPMP fueron casos sintomáticos, ya que el 33,3% fueron casos leves, el 53,3% moderados y el 13,3% severos (p<0,001). Por el contrario, no se encontró alguna relación estadísticamente significativa entre las variables de control del estudio y la RPMP (Tabla 1).

En el modelo crudo del análisis multivariado se encontró que, respecto a los casos asintomáticos, los casos leves (RPc=1,09; IC95%: 1,02‒1,17) y moderados/severos (RPc=1,63; IC95%: 1,40‒1,88) tuvieron una mayor probabilidad de presentar RPMP. Esta asociación se mantuvo en el modelo multivariado ajustado por anemia durante el embarazo, infecciones ascendentes durante el embarazo, sangrado vaginal durante el embarazo, control prenatal adecuado, número de partos previos, antecedente de parto prematuro previo y antecedente de pérdida fetal, donde los casos leves tuvieron 1,10 veces la probabilidad de tener RPMP (RPa=1,10; IC95%: 1,02‒1,18) y los casos moderados/severos tuvieron 1,64 veces la probabilidad (RPa=1,64; IC95%: 1,43‒1,87) en comparación con los casos asintomáticos (Tabla 2).

**Tabla 2:** Asociación entre el grado de severidad de la infección por COVID-19 durante el embarazo y la rotura prematura de membranas pretérmino: modelos crudo y ajustado.

Variables	RPc	IC95%	Valor de p	RPa^a^	IC95%	Valor de p
Grado de severidad de la infección por COVID-19 durante el embarazo						
Caso asintomático	Ref	-	-	Ref	-	-
Caso leve	1,09	1,02‒1,17	0,015	1,10	1,02‒1,18	0,012
Caso moderado o severo	1,63	1,40‒1,88	<0,001	1,64	1,43‒1,87	<0,001

RPc: razón de prevalencia cruda; RPa: razón de prevalencia ajustada; IC95%: intervalo de confianza al 95%.

a Modelo ajustado por: anemia durante el embarazo, infecciones ascendentes durante el embarazo, sangrado vaginal durante el embarazo, control prenatal adecuado, número de partos previos, antecedente de parto prematuro previo y antecedente de pérdida fetal.

## DISCUSIÓN

Los principales resultados del presente estudio demuestran que cerca de una de cada diez pacientes infectadas por COVID-19 desarrollaron RPMP. Todas ellas correspondieron a casos sintomáticos. Las gestantes con casos leves tuvieron 1,10 veces la probabilidad de presentar RPMP y las que presentaron casos moderados/severos tuvieron 1,64 veces la probabilidad comparada con las pacientes asintomáticas.

La frecuencia de la RPMP encontrada es similar a la descrita en otros estudios. En un metaanálisis que incluyó 16 estudios transversales y serie de casos, se encontró que la frecuencia de RPMP en mujeres embarazadas e infectadas con el SARS-CoV-2 fue de 9,9% ^8^. Otra revisión sistemática de 39 estudios, en su mayoría retrospectivos y serie de casos, mostró que la RPMP ocurrió en el 8,9% de embarazos con infección por el SARS-CoV-2 ^23^. Por el contrario, en una revisión sistemática de 19 estudios, principalmente reportes de casos y retrospectivos, se evidenció que la RPMP ocurrió en 20,7% de las pacientes infectadas por el virus ^24^. Esta mayor frecuencia respecto a los resultados del presente estudio podría deberse a que todas las gestantes que se incluyeron se encontraban hospitalizadas con signos de severidad, y el intervalo de confianza de la frecuencia mencionada fue amplio, lo que indicaría una estimación menos precisa ^24^.

Los resultados sugieren que la severidad de la infección por COVID-19 está asociada a mayor probabilidad de complicaciones durante el embarazo, tal como han demostrado otras investigaciones. En un estudio que incluyó 1219 gestantes, se encontró que los resultados perinatales adversos eran más comunes en pacientes con una enfermedad por COVID-19 más severa ^17^. Aquellas pacientes con un cuadro severo/crítico tuvieron un riesgo incrementado de cesárea, trastornos hipertensivos del embarazo y parto pretérmino en comparación con las pacientes asintomáticas ^17^. De igual manera, en una revisión sistemática de 62 estudios, en su mayoría de cohortes, se encontró que aquellas pacientes con la COVID-19 severa tuvieron mayor riesgo de desarrollar diabetes gestacional, preeclampsia, RPM (incluida RPMP), restricción de crecimiento intrauterino, distrés fetal y desprendimiento prematuro de placenta ^18^. En Perú, una investigación con resultados similares encontró que la presencia de síntomas de infección por COVID-19 llevó a un riesgo aumentado de complicaciones maternas como variable compuesta, que incluyó la RPMP, que estuvo presente en 25,9% de casos sintomáticos y solo en 9,5% de asintomáticos ^19^. A pesar de estas semejanzas, este estudio evalúa específicamente la asociación de la RPMP, como variable única, con todos los grados de severidad de la infección, no solo los casos severos, lo que constituye un aporte al conocimiento de este tema.

El desarrollo de RPMP tras la infección por COVID-19 puede ser explicado por la presencia de cambios inflamatorios en las membranas corioamnióticas, incluida la placenta, que suelen preceder el desarrollo de RPMP y que representan hallazgos frecuentemente encontrados en gestantes con la COVID-19 ^11^. El ingreso del virus SARS-CoV-2 a las células humanas se produce a través del receptor ACE-2, presente también en el tejido placentario, donde se ha podido encontrar partículas virales e infiltración de células inflamatorias, en su mayoría monocitos y neutrófilos ^25^. De igual manera, algunos estudios han demostrado que la infección por el SARS-CoV-2 puede llevar a cambios histopatológicos significativos en la placenta, siendo los más comunes malformaciones vasculares maternas y fetales, con perfusión placentaria deficiente; formación de trombos y vilitis ^25^^,^^26^. En ese sentido, una investigación encontró que el 25% de las placentas de mujeres infectadas eran pequeñas para la edad gestacional y el 77% presentaban características de mala perfusión ^27^. Estos hallazgos sugieren que la COVID-19 puede ocasionar un desarrollo inadecuado de la placenta, predisponiendo a las gestantes a desenlaces maternos y fetales desfavorables, incluida la RPMP ^28^.

La relación con la severidad de la enfermedad puede explicarse por cierta evidencia que demuestra que las concentraciones de anticuerpos anti-SARS-CoV-2 (anti-Spike), que desencadenan la reacción inflamatoria en respuesta a la presencia del virus, es mayor en gestantes con una enfermedad moderada a severa/crítica ^28^. De esta manera, el mayor desarrollo de anormalidades placentarias es directamente proporcional a la concentración de anticuerpos y severidad de la COVID-19, observadas en el 57% de los casos asintomáticos/leves, a diferencia del 100% de los casos moderados a severos ^28^. Asimismo, la presencia de cambios placentarios de mayor gravedad, incluida la necrosis, fueron descritos en pacientes que requirieron oxígeno suplementario o intubación, es decir, casos más severos ^27^.

En el ámbito de salud pública, estos resultados reafirman la necesidad de priorizar la atención de gestantes según el grado de severidad de la infección por COVID-19 para poder brindarles un tratamiento oportuno. Asimismo, justifica las decisiones administrativas que prioricen el destino de recursos hacia la atención de gestantes con la enfermedad. De igual forma, los datos recopilados pueden ser utilizados como un estudio preliminar para futuras investigaciones vinculadas al tema de estudio, sobre todo tomando en consideración que los resultados podrían cambiar en el contexto de una realidad actual con alta tasa de vacunación contra la COVID-19. En efecto, una revisión sistemática de nueve estudios encontró que la vacunación en gestantes reduce la probabilidad de partos prematuros por diversas causas, incluida la RPMP ^29^.

El presente estudio cuenta con algunas limitaciones. Debido a que no se encontraron investigaciones que hayan evaluado la prevalencia de RPMP según cada grado de severidad de la infección por COVID-19 en el momento de diseñar el protocolo, para el cálculo de la muestra se utilizaron estudios con proporciones parecidas. Además, dado que el número de participantes terminó correspondiendo al tamaño mínimo de muestra calculado y que existe una desproporción entre el número de casos asintomáticos (n=92) y los moderados/severos (n=16), su comparación podría constituir un sesgo clínico. No obstante, debido al carácter exploratorio del estudio, se considera que la muestra es suficiente para plantear hipótesis que deben ser verificadas en investigaciones posteriores. De igual manera, podría existir un sesgo de selección debido a que se trató de un estudio unicéntrico, sin embargo, el hospital elegido es un centro de referencia nacional que acoge gestantes de diversas partes del Perú. Existen otros factores predisponentes para la RPMP que no se evaluaron en este estudio porque corresponden a información que no suele encontrarse en las historias clínicas, como la presencia de deficiencias nutricionales o antecedente de RPMP previa. Por esa misma razón, otros datos analíticos, como la edad gestacional al diagnóstico de COVID-19 y la edad gestacional al momento de la RPMP, no pudieron ser incluidos. Además, las historias clínicas se encuentran escritas a mano, de una manera no sistematizada y propensa a errores, por lo que se debe confiar que la información haya sido completada de forma adecuada. Asimismo, existe la posibilidad de que algunas pacientes atendidas al inicio de la pandemia no hayan sido correctamente diagnosticadas con la enfermedad por déficit de pruebas diagnósticas, de manera que puede existir un subregistro de gestantes con la COVID-19 y RPMP. Por último, actualmente la enfermedad por COVID-19 ha evolucionado a un contexto donde existe vacunación y control de la emergencia sanitaria, lo que podría tener un efecto sobre la interpretación actual de los hallazgos.

En conclusión, se encontró que un mayor grado de severidad de la infección por COVID-19 durante el embarazo se asocia a una mayor probabilidad de desarrollar RPMP. Debido al carácter exploratorio del estudio, se recomendaría realizar investigaciones análogas posteriores, utilizando un mayor tamaño de muestra y representatividad, y tomando en consideración el efecto de la vacunación y el contexto sanitario actual. No obstante, se considera que el presente estudio provee resultados que, debido a su plausibilidad, amerita que se tomen en cuenta en la toma de decisiones al evaluar a una paciente infectada por COVID-19.
